# Gimatecan exerts potent antitumor activity against gastric cancer in vitro and in vivo via AKT and MAPK signaling pathways

**DOI:** 10.1186/s12967-017-1360-z

**Published:** 2017-12-13

**Authors:** Zuhua Chen, Zhentao Liu, Wenwen Huang, Zhongwu Li, Jianling Zou, Jingyuan Wang, Xiaoting Lin, Beifang Li, Dongshao Chen, Yanting Hu, Jiafu Ji, Jing Gao, Lin Shen

**Affiliations:** 1 Key laboratory of Carcinogenesis and Translational Research (Ministry of Education), Department of Gastrointestinal Oncology, Peking University Cancer Hospital & Institute, Fu-Cheng Road 52, Hai-Dian District, Beijing, 100142 China; 2 Key laboratory of Carcinogenesis and Translational Research (Ministry of Education), Department of Pathology, Peking University Cancer Hospital & Institute, Beijing, China; 3 Key laboratory of Carcinogenesis and Translational Research (Ministry of Education), Department of Gastrointestinal Surgery, Peking University Cancer Hospital & Institute, Beijing, China

**Keywords:** Gimatecan, Patient-derived xenografts, Gastric cancer, MAPK pathway

## Abstract

**Background:**

We investigated antitumor activity and underlying mechanisms of DNA topoisomerase I (TopI) inhibitor gimatecan and irinotecan in gastric cancer (GC) in vitro cell lines and in vivo patient-derived xenograft (PDX) models.

**Methods:**

GC cell lines SNU-1, HGC27, MGC803 and NCI-N87 were used to evaluate cell viability and apoptosis after gimatecan or irinotecan treatment, using a cell proliferation assay and flow cytometry, respectively. DNA TopI expression and critical molecules of PI3K/AKT, MAPK and apoptosis signaling pathways were analyzed with western blot. For in vivo studies, five PDXs models were treated with gimatecan or irinotecan to assess its antitumor activity. Immunohistochemistry staining of Ki-67 was performed after mice were sacrificed.

**Results:**

Gimatecan inhibited the proliferation of GC cells in vitro in a dose- and time-dependent manner by inducing apoptosis, and gimatecan had greater inhibitory effects than irinotecan. In addition, both gimatecan and irinotecan demonstrated significant tumor growth inhibition in in vivo PDX models. Gimatecan treatment significantly inhibited the expression of DNA TopI, phosphorylated AKT (pAKT), phosphorylated MEK (pMEK) and phosphorylated ERK (pERK). Meanwhile, gimatecan could also activate the JNK2 and p38 MAPK pathway as indicated by upregulation of phosphorylated p38 MAPK (p-p38) and phosphorylated JNK2 (pJNK2).

**Conclusions:**

For the first time, we have shown that the antitumor activity of gimatecan in GC via suppressing AKT and ERK pathway and activating JNK2 and p38 MAPK pathway, which indicated that gimatecan might be an alternative to irinotecan in the treatment of GC.

**Electronic supplementary material:**

The online version of this article (10.1186/s12967-017-1360-z) contains supplementary material, which is available to authorized users.

## Background

In China, gastric cancer (GC) is the second leading cause of cancer-related deaths, and about 80% of patients with GC are diagnosed at an advanced stage [1, 2]. Typically, chemotherapy is the cornerstone of treatment for advanced gastric cancer (AGC). Despite the fact that the combination of fluorouracil-based chemotherapy and trastuzumab has provided HER2-positive patients with significant survival benefit [3], prognosis for patients with AGC is still grave due to the limited treatment options and inevitable drug resistance. Therefore, exploring potential novel drugs is needed for GC.

Camptothecin (CPT) is a pentacyclic quinoline alkaloid isolated from the Chinese *Camptotheca acuminata* tree. Based on the CPT structure, 10-hydroxycamptothecin (HCPT), irinotecan, topotecan, gimatecan and other analogues have been developed as broad-spectrum antitumor drugs to treat colorectal cancer [4], lung cancer [5], melanoma [6], hepatic carcinoma [7] and neuroblastoma [8]. The direct target of CPT and its derivatives is DNA topoisomerase I (TopI), which breaks DNA by bonding to 3′-phosphates [9]. TopI is susceptible to inhibitors when DNA is in a cleaved state, allowing inhibitors to convert transient TopI-DNA complexes to permanently damaged strands. These inhibitors have weak affinities for the enzyme or DNA alone [10]. In addition to negative regulation of TopI, HCPT has been reported to enhance apoptosis via p53 [8], p38 MAPK, ERK, AKT [11], and NF-κB [12] pathways.

Gimatecan, which is an orally bioavailable CPT analogues and has greater and more persistent DNA cleavage than other CPTs [13–15], has been shown to have strong preclinical antitumor activity against a panel of human tumor xenografts [16–20]. Furthermore, a phase I study in 33 patients with advanced solid tumors confirmed the antitumor activity and acceptable tolerability of gimatecan, which warrants further clinical researches to evaluate the efficacy of gimatecan monotherapy or combination with other agents [21–24]. Irinotecan is frequently used in GC patients as second- or third-line therapy, but whether gimatecan has antitumor activity against GC is unclear.

## Methods

### Cell lines

Human GC cell lines SNU-1, HGC27 and MGC803 were purchased from Peking Union Medical College, and the NCI-N87 cell line was a gift from You-yong Lv, Ph.D. (Peking University Cancer Hospital and Institute). Cells were cultured in RPMI 1640 medium and Dulbecco’s Modified Eagle Medium (Gibco-BRL, MD, USA), respectively, supplemented with 10% fetal bovine serum (Gibco-BRL), 100 U/ml penicillin (Gibco-BRL) and 100 mg/ml streptomycin (Gibco-BRL). Cells were incubated in a humidified incubator (37 °C) supplemented with 5% CO_2_.

### Inhibitors and antibodies

Gimatecan (purity ≥ 99.9%) was provided by Zhaoke Pharmaceutical Ltd. (Hefei, China), and irinotecan hydrochloride (purity = 99.91%) was purchased from Jiangsu Hengrui Medicine Co., Ltd. (Jiangsu, China). Gimatecan was dissolved in DMSO at a stock concentration of 10 mmol/l and 12.5 mg/ml for in vitro and in vivo studies, respectively, and then stored at − 80 °C for future use. Irinotecan was diluted in 0.9% NaCl at a concentration of 10 mmol/l and 20 mg/ml immediately before use. AKT, pAKT, S6, pS6, ERK, pERK, MEK, pMEK, p38 MAPK, p-p38 MAPK, JNK2, pJNK2, Bcl-2, Bak, PARP, cleaved PARP, MDR1, ABCG2 and DNA Topoisomerase I antibodies were purchased from Cell Signaling Technology (Boston, MA, USA). β-Actin antibody was purchased from Sigma-Aldrich (St. Louis, Missouri).

### Cell viability assay

SNU-1, HGC27, MGC803 and NCI-N87 cells (5000 cells/well) were seeded in 96-well plates and incubated overnight in complete medium, followed by exposure to gimatecan (0–1 µM) or irinotecan (0–1 µM) for 24, 48, or 72 h. Cell viability was measured using a Cell Counting Kit-8 (Dojindo, Kumamoto, Japan) according to the manufacturer’s instructions. Absorbance at 450 nm was measured using a microplate spectrophotometer. All experiments were repeated at least three times.

### Annexin V apoptosis assay

Apoptosis was measured by staining with phycoerythrin (PE)-annexin V and 7-amino-actinomycin (7-AAD) (BD Biosciences, Erembodegem, Belgium) for 15 min at room temperature in the dark, followed by flow cytometry (BD Biosciences) within 1 h. Apoptosis was analyzed with FlowJo 7.6 software (FlowJo, LLC, Ashland, Oregon).

### Animal experiments

Establishment and serial passaging of GC patient-derived xenografts (PDX) models were as previously described [25]. All procedures were performed under sterile conditions at an SPF facility and carried out in accordance with the guide for the Care and Use of Laboratory Animals of the National Institutes of Health. Animal experiments were approved by an independent ethics committee of Peking University Cancer Hospital.

Five PDX tissues were subcutaneously inoculated into the flanks of 6-week-old female non-obese diabetic/severe combined immunodeficiency (NOD/SCID) mice. When tumors reached 150–250 mm^3^, mice were randomized to three groups (N = 5/group) with similar tumor volumes: (1) control group (physiological saline 100 µl daily, by orally gavage), (2) gimatecan group (gimatecan 0.2 mg/kg daily, by orally gavage) and (3) irinotecan group (irinotecan 20 mg/kg via weekly intraperitoneal injection). All animals were treated for 3 weeks. Tumor size and body weight were measured twice a week, and tumor volume was calculated using the following formula: V = (L × W^2^)/2 (V, volume; L, length; W, width). Tumor growth inhibition (TGI) was calculated using the following formula: TGI = 1 − ΔT/ΔC × 100% (ΔT = tumor volume changes of the drug treated group, ΔC = tumor volume changes of the control group on the final day of the study).

### Western blot

SNU-1, HGC27 and NCI-N87 cells were starved in serum-free medium overnight, exposed to inhibitors for 48 h and harvested at 70–80% confluence. Total protein was extracted from cells or xenograft tissues on ice, using RIPA Lysis Buffer (Beyotime, Shanghai, China) supplemented with complete protease inhibitor and phosphatase inhibitor cocktail (Roche, Basel, Switzerland). Protein concentration was measured using a BCA Protein Assay Kit (Beyotime, Shanghai, China), and 50 μg protein from each sample was separated by 10% SDS-PAGE. After transfer to nitrocellulose membranes (GE Healthcare, Piscataway, NJ), samples were incubated with corresponding primary antibodies diluted in 5% BSA at 4 °C overnight and then with secondary antibodies at room temperature for 1 h. Proteins were visualized with a chemiluminescent detection system (GE Healthcare), using ECL plus Western blot reagents (GE Healthcare). Western-blotting bands were quantified and normalized by ImageJ software.

### Immunohistochemistry (IHC) staining of Ki-67

After mice were sacrificed, xenograft tissues were isolated, and formalin-fixed paraffin embedded (FFPE) tissue sections were prepared. After deparaffinization, hydration, endogenous peroxidase treatment, and retrieval, 4-μm-thick FFPE sections were incubated with primary antibodies (1:100) overnight at 4 °C. The signal was assayed after incubation with IgG-HRP polymer (ZSGB-BIO, Beijing, China) and diaminobenzidine substrate. Sections were interpreted by pathologists from the Department of Pathology of Peking University Cancer Hospital who were blinded to this study. The scoring standard of Ki-67 was consistent with a previous report [26].

### Statistical analysis

Statistical analysis was performed with Graphpad Prism version 6.0 (Graphpad software). For in vitro studies, differences between the groups were analyzed using an unpaired two-tailed t test. For in vivo studies, tumor growth among different groups was compared using repeated measures ANOVA and *p* < 0.05 was considered statistically significant.

## Results

### Gimatecan inhibited cell proliferation in a dose- and time-dependent manner in vitro

**Fig. 1:**
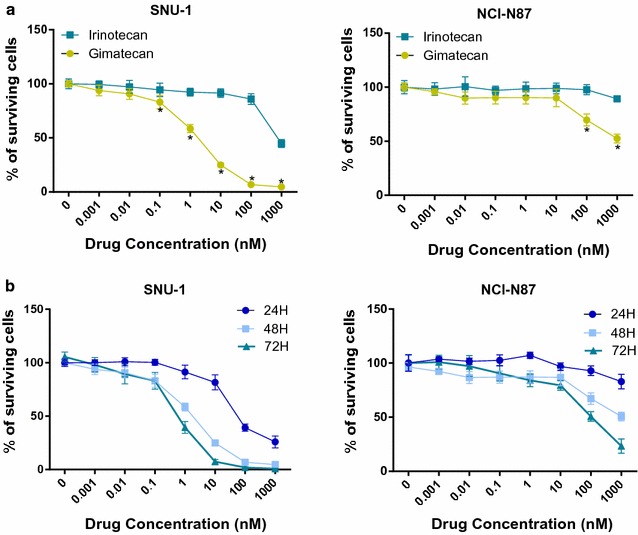
Gimatecan inhibits proliferation of human GC cells. **a** Gimatecan demonstrated inhibitory effects on SNU-1 and NCI-N87 cells, as well as irinotecan. SNU-1 and NCI-N87 cells were seeded in 96-well plates and incubated overnight in complete medium, followed by exposure to gimatecan (0–1 µM) or irinotecan (0–1 µM) for 48 h. The surviving cells were evaluated by CCK-8 assay. **b** Gimatecan inhibited proliferation of human GC cells in a dose and time dependent manner. SNU-1 and NCI-N87 cells were treated with gimatecan (0–1 µM) for 24, 48 and 72 h. All data were presented as mean ± SD of three independent experiments. *p < 0.05

SNU-1, HGC27, MGC803 and NCI-N87 cell lines were treated with gradient dilutions of gimatecan and irinotecan for 24, 48, and 72 h. Compared with irinotecan, gimatecan had superior antiproliferative effects on SNU-1 (IC50 1.95 nM vs. 3253.71 nM, *p* < 0.05), HGC27 (IC50 1.63 nM vs. 151.90 nM, *p* < 0.05), MGC803 (IC50 3.29 nM vs. 429,205.00 nM, *p* < 0.05) and NCI-N87 (IC50 88.20 nM vs. 141.90 nM, *p* < 0.05) cells (Fig. 1a, Additional file 1: Figure S1A, B). Furthermore, the antitumor activities of gimatecan were time-dependent, especially in the SNU-1 cell line (Fig. 1b). In addition, gimatecan was reported to be transported by chemoresistance-related proteins ABCG2 and MDR1, which could affect therapeutic response to gimatecan. So we evaluated the expression of ABCG2 and MDR1 in NCI-N87, SNU-1, MGC803 and HGC27 cell lines and found ABCG2 and MDR1 were highly expressed in NCI-N87 cells (Additional file 1: Figure S1C).

### Gimatecan induced apoptosis in GC

**Fig. 2:**
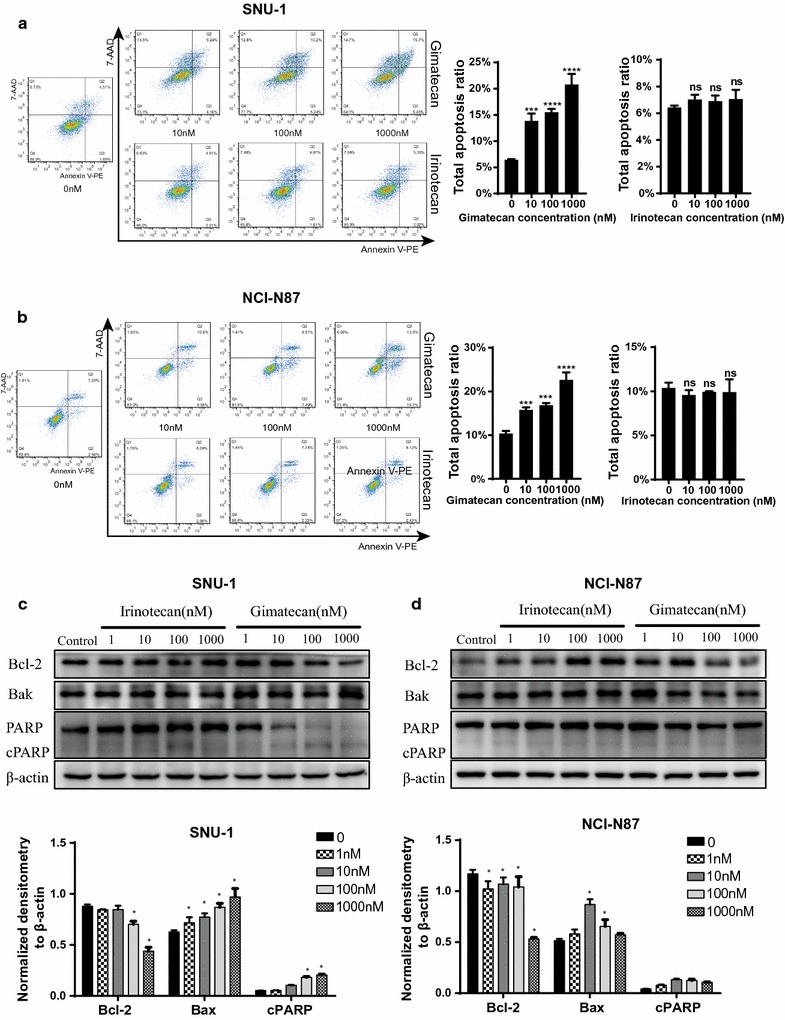
Gimatecan induces apoptosis in human GC cells. **a**, **b** Gimatecan, rather than irinotecan, significantly induced cell apoptosis in SNU-1 and NCI-N87 cells by flow cytometry assays. Cells were treated with gimatecan (0–1 µM) and irinotecan (0–1 µM) for 24 h and stained with Annexin V-PE/7-AAD. Sums of percentages of early apoptosis (Q3) and late apoptosis (Q2) were calculated as the total apoptosis ratios. **c**, **d** Pro- and anti-apoptotic proteins including Bcl-2, Bak, PARP and cleaved PARP were assessed by western-blotting in SNU-1 and NCI-N87 cells. Western-blotting bands were quantified and normalized by ImageJ. All data are mean ± SD of three independent experiments. *Compared with controls, *p < 0.05; ***p < 0.001; ****p < 0.0001

Apoptosis arises when cell growth is inhibited, so we measured this after gimatecan and irinotecan treatment for 24 h. Compared with controls, gimatecan treatment significantly increased the proportion of apoptotic cells in SNU-1 (14.38 ± 2.11% vs. 0.63 ± 0.65%, *p* < 0.05), HGC27 (14.27 ± 1.69% vs. 0.90 ± 0.17%, *p* < 0.05) and NCI-N87 cells (12.29 ± 2.24% vs. 0.47 ± 1.64%, *p* < 0.05) at the concentration of 1000 nM (Fig. 2a, b and Additional file 2: Figure S2A). Meanwhile, this effect was in a dose-dependent manner. However, apoptosis induced by irinotecan was not obvious compared with controls.

Western blot was used to measure Bak, Bcl-2, and PARP protein after gimatecan and irinotecan treatment. Consistent with flow cytometry data, Bak and cleaved PARP expression was increased and Bcl-2 expression was inhibited by gimatecan treatment in SNU-1 (Fig. 2c) and HGC27 (Additional file 2: Figure S2B), which confirmed apoptosis. However, we didn’t observe the same tendency in NCI-N87 after treatment of gimatecan (Fig. 2d). In contrast, apoptosis was not induced by irinotecan treatment.

### Gimatecan exerts antitumor activity via AKT and MAPK signaling pathways in vitro

**Fig. 3:**
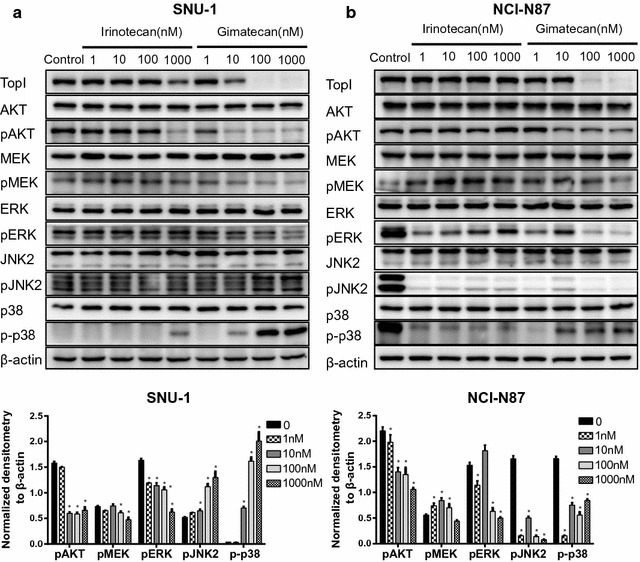
Gimatecan exerts antitumor activity via AKT and MAPK signaling pathways in vitro. **a** Gimatecan significantly inhibited the expression of TopI, pAKT, pMEK, and pERK, and activated the expression of p-p38 MAPK and pJNK2 in SNU-1 cells. **b** Gimatecan significantly inhibited the expression of pAKT and pERK in NCI-N87 cells. Cells were starved in serum-free medium overnight, exposed to gimatecan or irinotecan for 48 h and harvested at 70–80% confluence. Total protein of SNU-1 and NCI-N87 was extracted and the expression of TopI, pAKT, pMEK, pERK, p-p38 MAPK and pJNK2 were assessed by western-blotting followed by quantification and normalization by ImageJ. All data are mean ± SD of three independent experiments. Compared with controls, *p < 0.05

To investigate molecular events underlying gimatecan treatment, expressions of DNA TopI and molecules involved in AKT and MAPK pathways were quantified with western blot. Gimatecan treatment significantly inhibited expression of DNA TopI in SNU-1 (Fig. 3a), HGC27 (Additional file 3: Figure S3A) and NCI-N87 (Fig. 3b) cell lines at 100 nM or more, but irinotecan had little effect even at high concentrations. Moreover, gimatecan significantly inhibited the AKT pathway and activated the JNK2 and p38 MAPK pathway, as indicated by inhibition of pAKT, pMEK, and pERK, and upregulation of phosphorylated p38 MAPK (p-p38) and phosphorylated JNK2 (pJNK2), respectively, in SNU-1 (Fig. 3a) and HGC27 (Additional file 3: Figure S3B) cells. However, in NCI-N87 cells, gimatecan treatment only inhibited expression of pAKT and pERK (Fig. 3b). Our results indicated that SNU-1 and HGC27 cells were more sensitive to gimatecan than NCI-N87 cells, which was consistent with the result of cell viability.

### Gimatecan exerts antitumor activity via AKT and MAPK signaling pathways in vivo

**Fig. 4:**
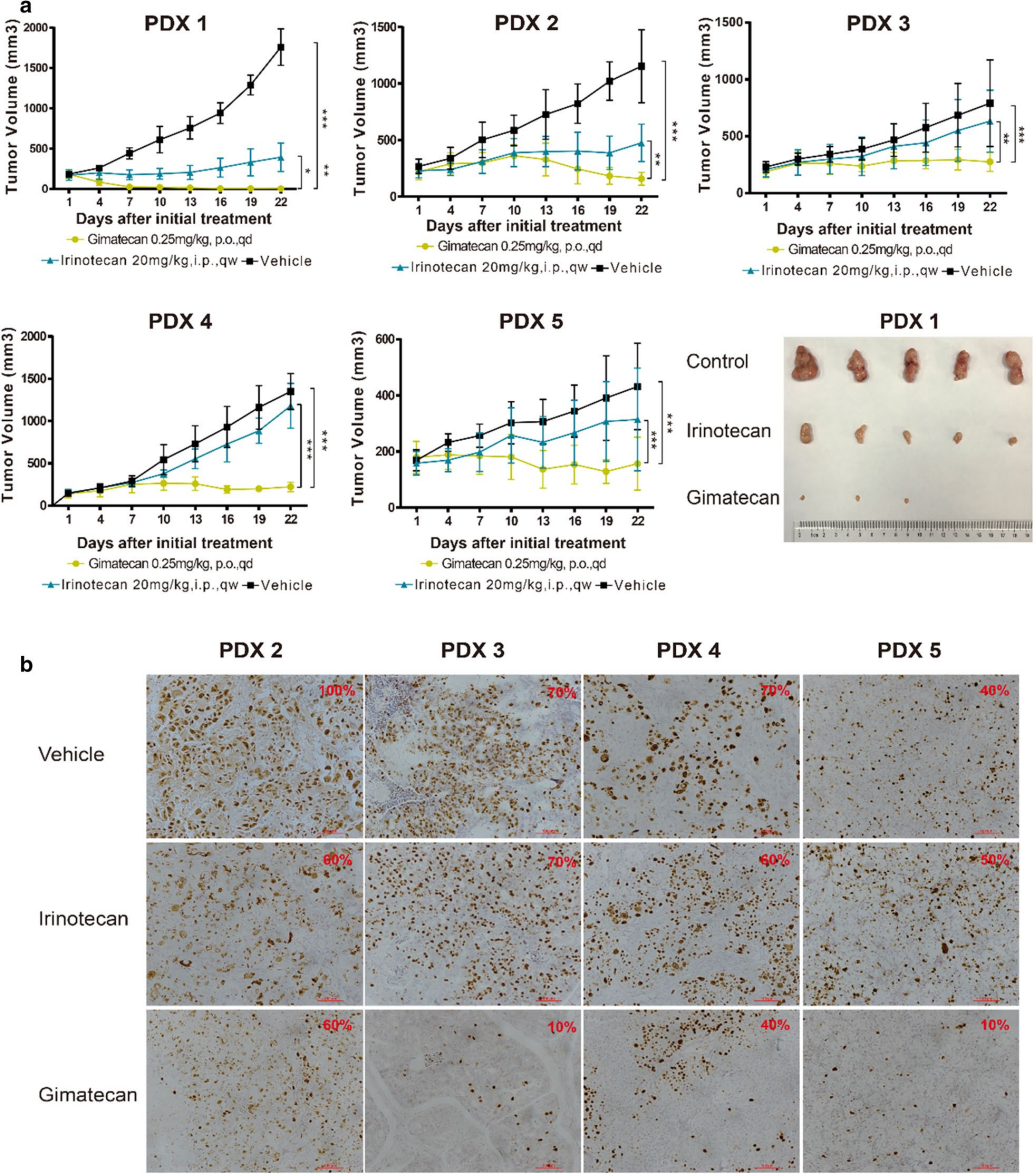
Gimatecan inhibited tumor growth of xenografts on PDX models in vivo. **a** Gimatecan significantly inhibited tumor growth in xenografts from all five PDX models. A PDX model was established by subcutaneously transplanting tumor tissues of a patient into NOD/SCID mice and PDX 1–5 indicated PDX models from 5 different GC patients. PDX tissues were subcutaneously inoculated into mice, and when the tumor size reached 150–250 mm^3^, mice (n = 5 in each group) were treated with buffer control or inhibitors. Tumor volumes were presented as mean ± SD. The antitumor activity is depicted by tumor growth inhibition (TGI). TGI = ΔT/ΔC × 100% (ΔT = tumor volume change of the drug-treated group, ΔC = tumor volume change of the control group on the final day of the study). *p < 0.05, **p < 0.01, ***p < 0.001. **b** Immunohistochemical staining for Ki-67 of xenografts on day 22 of treatment from four PDX models. The ki-67 index, marker of cell proliferation, was calculated as the proportion of positive tumor cell nuclei in all tumor cells examined and labeled in red in the images. Scale bar represents 100 µm

Preclinical PDX models were used to validate antitumor activity of gimatecan in vivo. Compared with control groups, both gimatecan and irinotecan showed significant antitumor activity in all xenografts (Fig. 4a) and TGI data appear in Table 1. Using Ki-67 staining, we found that gimatecan inhibited proliferation of xenograft tissues (Fig. 4b and Table 1), and proliferation was inversely correlated with inhibitory effects.

**Table 1:** The tumor growth inhibitions (TGIs) and Ki-67 scores after gimatecan treatment in five xenografts

No. of PDX model	TGIs (%)		Ki-67 (%)		
	Gimatecan	Irinotecan	Control	Gimatecan	Irinotecan
1	110.1	75.4	–^a^	–^a^	–^a^
2	106.6	76.1	100	60	60
3	84.4	24.1	70	10	70
4	91.7	15.1	70	40	60
5	108.7	40.6	40	10	50

^a^No tissue was available for immunohistochemistry assay after the treatment

**Fig. 5:**
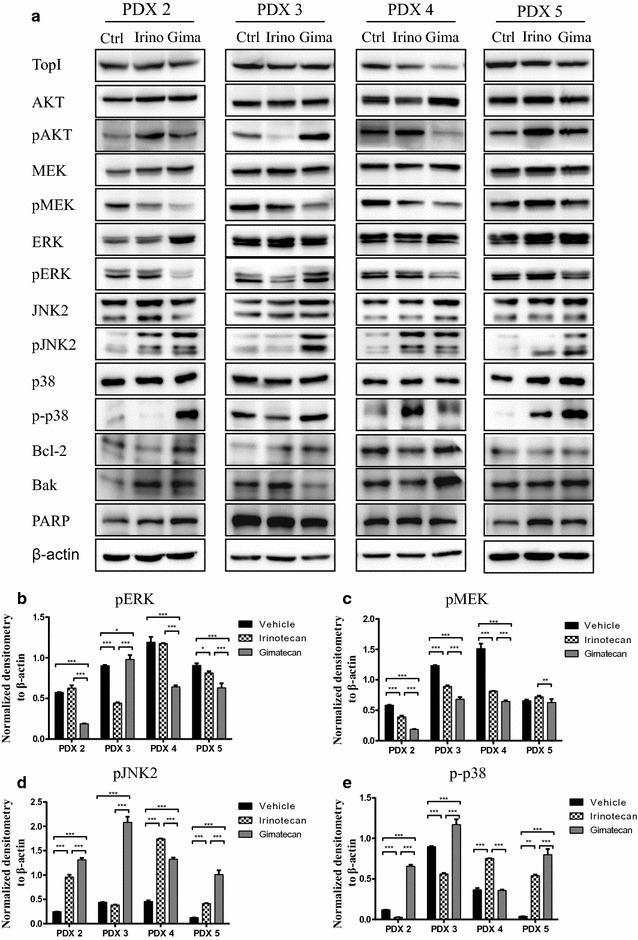
Gimatecan exerts antitumor activity via AKT and MAPK signaling pathways in vivo. **a** Western-blotting of critical molecules in AKT and MAPK signaling pathways in PDX tissues. **b**–**e** Quantification and normalization of western-blotting bands of pERK, pMEK, pJNK2 and p-p38. After mice were sacrificed, total protein was extracted from PDX tissues and the expression of critical molecules in AKT and MAPK signaling pathways were assessed by western-blotting. Gimatecan significantly inhibited expression of TopI, pMEK and pERK, and upregulated p-p38 MAPK and pJNK2. *p < 0.05, **p < 0.01, ***p < 0.001

Data show that AKT and MAPK signaling pathways may be involved in tumor suppression. After gimatecan treatment, pMEK and pERK expression were inhibited in some xenograft tissues and pJNK2 and p-p38 MAPK expressions was upregulated (Fig. 5). These data agree with in vitro studies and suggested that gimatecan has antitumor activity in vivo PDX models via AKT and MAPK pathways (Additional file 4: Figure S4).

## Discussion

In this study, we evaluated the efficacy and underlying mechanism of gimatecan and irinotecan in vitro and in vivo. Gimatecan had significant antitumor activity as indicated by inhibition of cell proliferation, suppression of xenograft growth, and activation of apoptosis.

As is known to us, TopIs have been described as molecular targets for CPT and its derivatives, and TopI is essential for DNA replication, recombination, and damage repair. Two water-soluble CPT derivatives have been approved by the FDA: topotecan for ovarian cancer and recurrent small cell lung cancer [10], and irinotecan for gastrointestinal cancer, which has been developed as a single agent or in combination with other cytotoxic agents for second- or third-line therapy for advanced AGC [27–30]. However, the instability of lactone ring and poor oral bioavailability have been reported to be major limitations of water-soluble CPT derivatives in clinical practice [31].

As the third orally bioavailable CPT analogue, gimatecan induced proliferative inhibition and apoptosis promotion in GC cells at a lower concentration, which was consistent with previous studies [13–18]. In 2007, Marchetti et al. reported that the ABCG2 expression resulted in eight to tenfold resistance to gimatecan, which could be reversed by the ABCG2 or MDR1 inhibitors [32]. In this study, we detected the expression of ABCG2 and MDR1 in four GC cell lines and observed higher expression of ABCG2 and MDR1 in NCI-N87 cell line, which might be the reason why gimatecan was relatively insensitive to NCI-N87 cells. Moreover, it was well known that most chemotherapeutics had effect on normal cells, therefore, our result also suggested that gimatecan could lead to weak growth inhibition in normal immortalized gastric epithelial cell line (data not shown). But even so, the inhibitory activity of gimatecan was still a promising strategy in the treatment of AGC.

Gimatecan has been reported to decrease expression and activity of TopI, and induce cell cycle arrest at the S phase via cytotoxicity [17]. Other potential molecular events such as upregulation of TRAIL-R1 and -R2 [33], inhibition of pAkt and induction of anti-angiogenesis [20] have been reported and efforts have been made to explore TopI mutations [15] and plasma alpha1-acid glycoprotein as biomarkers [34]. In the present study, we tried to elaborate the potential mechanism of mitochondria-dependent apoptosis induced by MAPK pathways.

As critical regulators of cell apoptosis, Bcl-2 family can be divided into pro-apoptotic protein such as Bak, Bad and Bid, and anti-apoptosis proteins including Bcl-2 and Bcl-xl. In our study, compared with irinotecan, gimatecan could induce obvious cell apoptosis accompanied by increased expression of Bak and decreased expression of Bcl-2 in SNU-1 and HGC27 cells. However, cell apoptosis was not significantly observed in xenograft tissues after gimatecan treatment, which might be mainly due to the tumor heterogeneity of xenografts.

Several studies suggest that mitogen-activated protein kinase (MAPK) and Akt signaling pathways respond to extra-cellular stimuli and are involved in apoptosis induced by CPT derivatives [11, 35]. In brief, the MAPK pathway consists of extracellular-signal-regulated kinase (ERK) which is associated with cell proliferation and growth, and the c-jun N-terminal kinase (JNK) and p38 MAPK pathways which are induced by cellular stress and are closely associated with cell death [36]. In this study, gimatecan can suppress phosphorylation of Akt and ERK, and increase expression of pJNK2 and p-p38 MAPK at a relatively low concentration in GC cells and PDXs. Inhibition of Akt and ERK signaling was consistent with antitumor activity of gimatecan in cells and in vivo xenografts. Meanwhile, activation of pJNK2 and p-p38 MAPK signaling confirmed cell death induced by a mitochondrial-dependent apoptosis pathway. However, we also found the activation of pJNK2 and p-p38 was inconsistent between SNU-1, HGC27 and NCI-N87 cells. This may partially result from the higher sensitivity of SNU-1 and HGC27 for gimatecan than NCI-N87. Besides, p38 and JNK2 were highly phosphorylated even under no treatment in NCI-N87 cells, which might be difficult to be further upregulated even under the treatment of gimatecan. This phenomenon also suggested the individual difference after the same treatment. Based on present results, we also proposed the hypothesis that p-p38 and pJNK2 levels might be predictive markers for gimatecan, which needed to be further investigated.

Our results indicated that gimatecan exerted significant antitumor activity in GC via suppressing AKT and ERK pathway and activating JNK2 and p38 MAPK pathway. Moreover, gimatecan is an orally bioavailable CPT analogue, whereas irinotecan is an intravenous formulation, suggesting that gimatecan might be an alternative to irinotecan and provided insight of gimatecan in the treatment of GC, which remained to be validated in further clinical research.

## Conclusions

Our current work is the first attempt to evaluate the antitumor effects of gimatecan in GC cell lines and PDX models, and this finding provides additional insights on the molecular events responsible for its anti-proliferative and antitumor potency.

## Additional files

**Additional file 1: Figure S1.** Gimatecan inhibits proliferation of human GC cells. (A) and (B) Gimatecan significantly inhibited cell proliferation in another human GC cell lines HGC27 and MGC803. Cells were seeded in 96-well plates and incubated overnight in complete medium, followed by exposure to gimatecan (0–1 µM) or irinotecan (0–1 µM) for 48 h. Cell viability was measured and presented as means ± SD of three independent experiments. *Compared with irinotecan at the same time, *p* < 0.05. (C) The expression of ABCG2 and MDR1 in four human GC cell lines.

**Additional file 2: Figure S2.** Gimatecan induces apoptosis in HGC27 cell line. (A) Compared with irinotecan, gimatecan induced higher cell apoptosis in HGC27 cell. Cells were treated with gimatecan (0–1 µM) and irinotecan (0–1 µM) for 24 h and stained with Annexin V-PE/7-AAD. Sums of percentages of early apoptosis (Q3) and late apoptosis (Q2) were calculated as the total apoptosis ratios. (B) Pro- and anti-apoptotic proteins including Bcl-2, Bak, PARP and cleaved PARP were assessed by western-blotting in HGC27 cell. Western-blotting bands were quantified and normalized by ImageJ. All data are means ± SD of three independent experiments. *Compared with controls, *p* *<* 0.05; ns, *p* > 0.05.

**Additional file 3: Figure S3.** Gimatecan exerts antitumor activity via AKT and MAPK signaling pathways in HGC27 cells. (A) Gimatecan significantly inhibited the expression of TopI, pAKT, pMEK, and pERK, and activated the expression of p-p38 MAPK and pJUNK2 in HGC27 cells. Cells were starved in serum-free medium overnight, exposed to gimatecan or irinotecan for 48 h and harvested at 70–80% confluence. Total protein of HGC27 was extracted and the expression of TopI, pAKT, pMEK, pERK, p-p38 MAPK and pJNK2 were assessed by western-blotting followed by quantification and normalization by ImageJ. All data are means ± SD of three independent experiments. Compared with controls, *, p < 0.05.

**Additional file 4: Figure S4.** Schematic representation of proposed pro-apoptotic signaling pathways triggered by gimatecan in GC.
