# Supplementary figures and images for: Gimatecan exerts potent antitumor activity against gastric cancer in vitro and in vivo via AKT and MAPK signaling pathways
Source: J Transl Med. 2017 Dec 13;15:253. doi: 10.1186/s12967-017-1360-z (PMC5729429; doi:10.1186/s12967-017-1360-z)

**A****HGC27**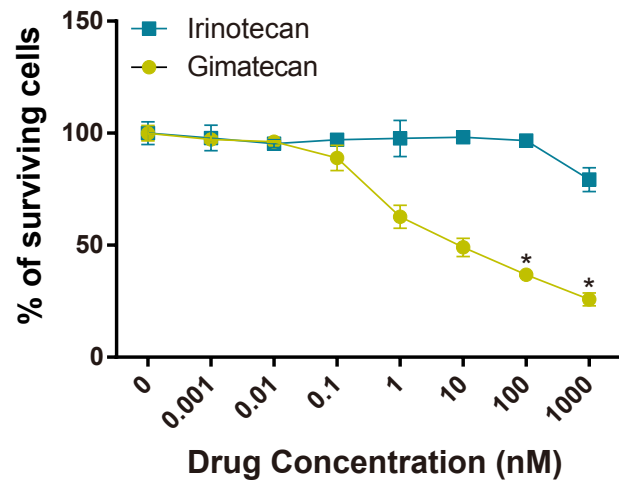**B****MGC803**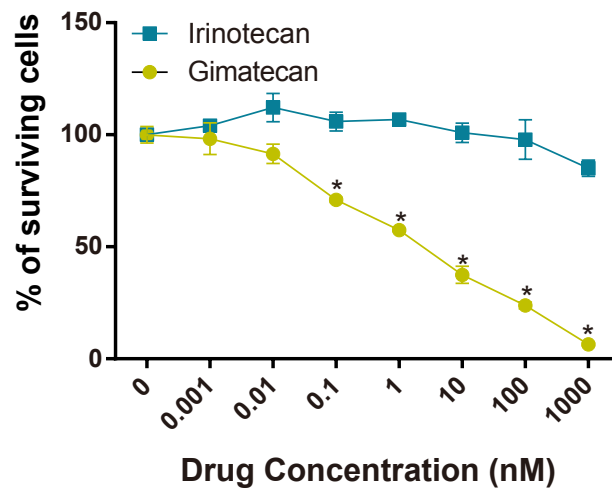**C**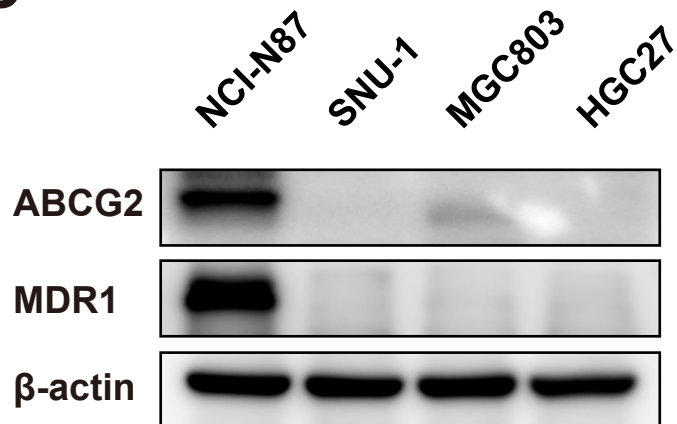

Supplement: Supplementary file 1 — Additional file 1: Figure S1. Gimatecan inhibits proliferation of human GC cells. (A) and (B) Gimatecan significantly inhibited cell proliferation in another human GC cell lines HGC27 and MGC803. Cells were seeded in 96-well plates and incubated overnight in complete medium, followed by exposure to gimatecan (0–1 µM) or irinotecan (0–1 µM) for 48 h. Cell viability was measured and presented as means ± SD of three independent experiments. *Compared with irinotecan at the same time, p < 0.05. (C) The expression of ABCG2 and MDR1 in four human GC cell lines. [file 12967_2017_1360_MOESM1_ESM.pdf]

**A****HGC27**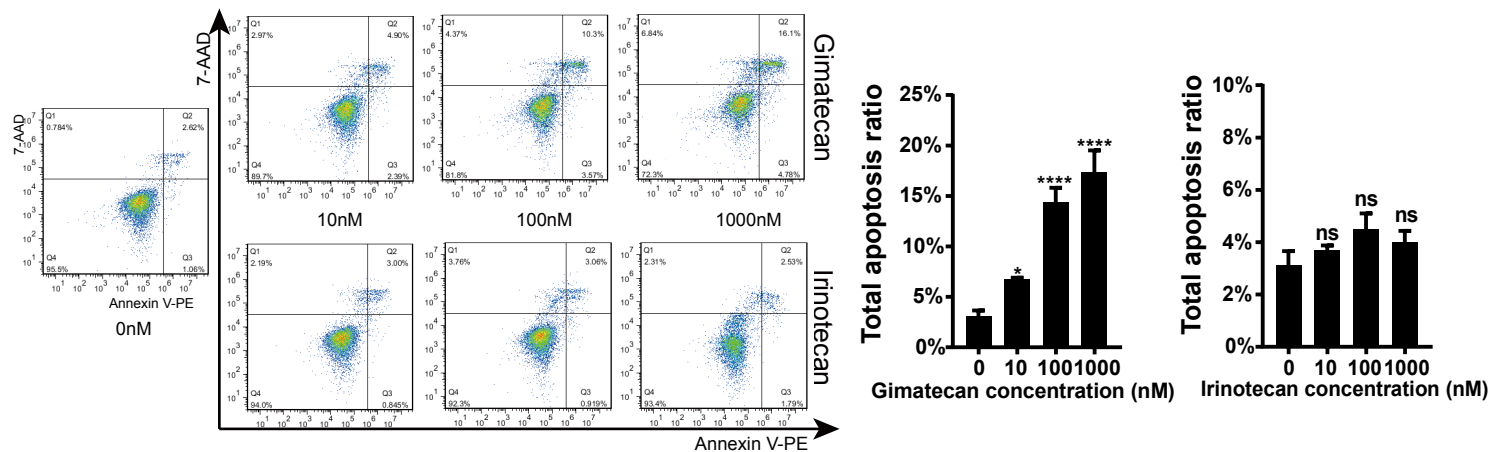**B**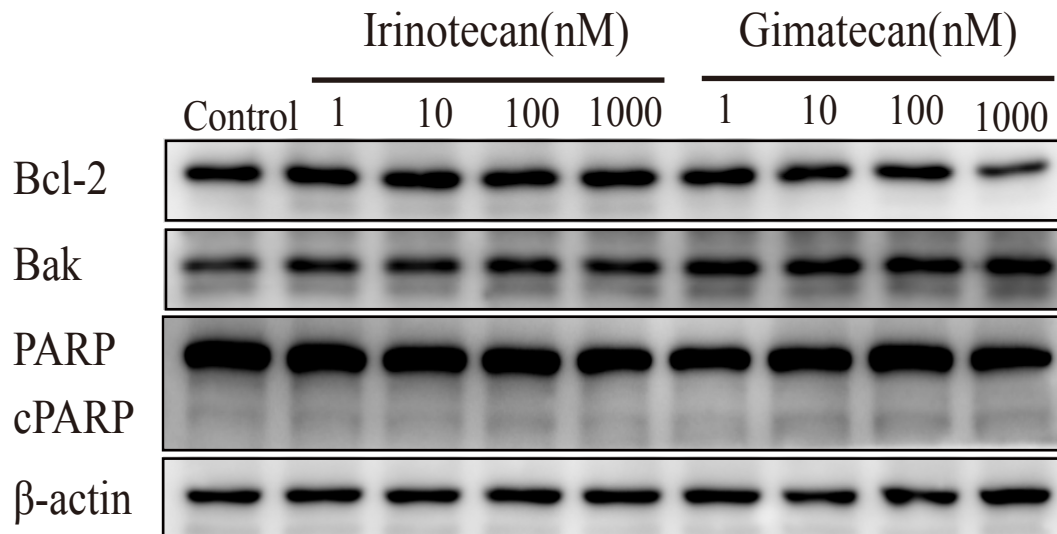

Supplement: Supplementary file 2 — Additional file 2: Figure S2. Gimatecan induces apoptosis in HGC27 cell line. (A) Compared with irinotecan, gimatecan induced higher cell apoptosis in HGC27 cell. Cells were treated with gimatecan (0–1 µM) and irinotecan (0–1 µM) for 24 h and stained with Annexin V-PE/7-AAD. Sums of percentages of early apoptosis (Q3) and late apoptosis (Q2) were calculated as the total apoptosis ratios. (B) Pro- and anti-apoptotic proteins including Bcl-2, Bak, PARP and cleaved PARP were assessed by western-blotting in HGC27 cell. Western-blotting bands were quantified and normalized by ImageJ. All data are means ± SD of three independent experiments. *Compared with controls, p < 0.05; ns, p > 0.05. [file 12967_2017_1360_MOESM2_ESM.pdf]

**A**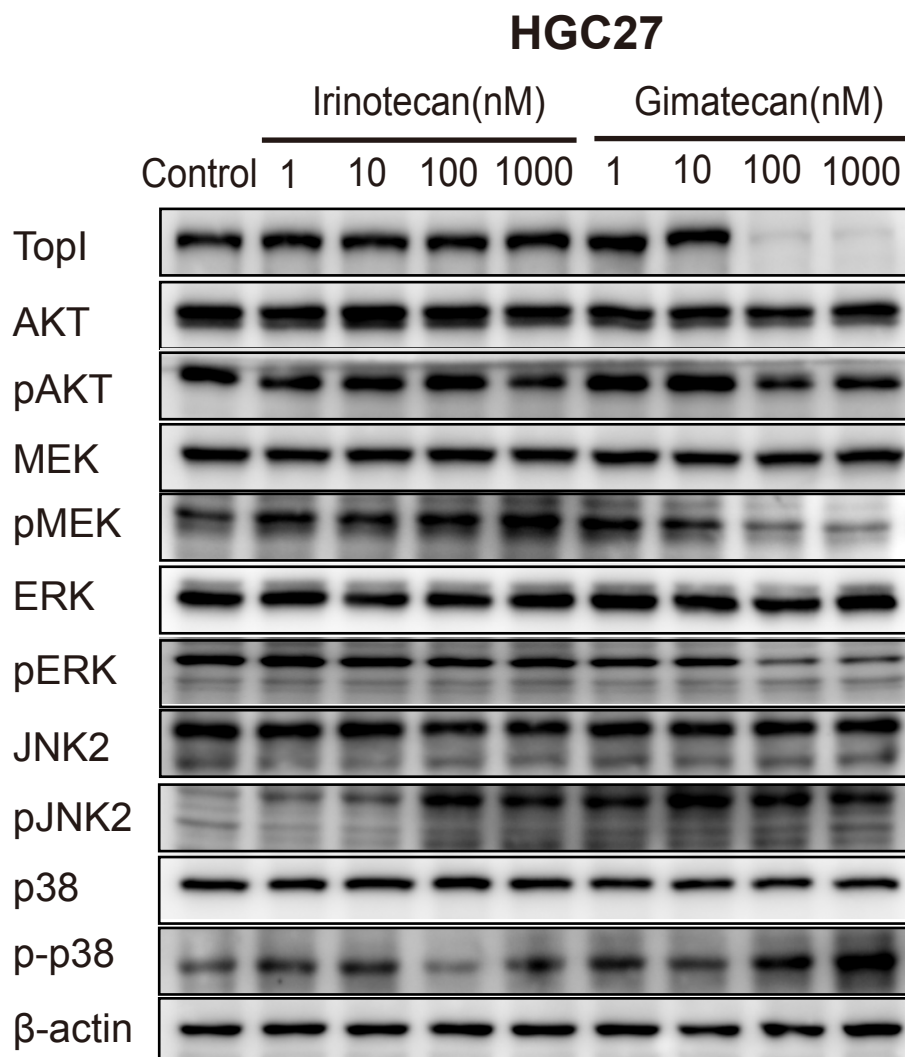**B**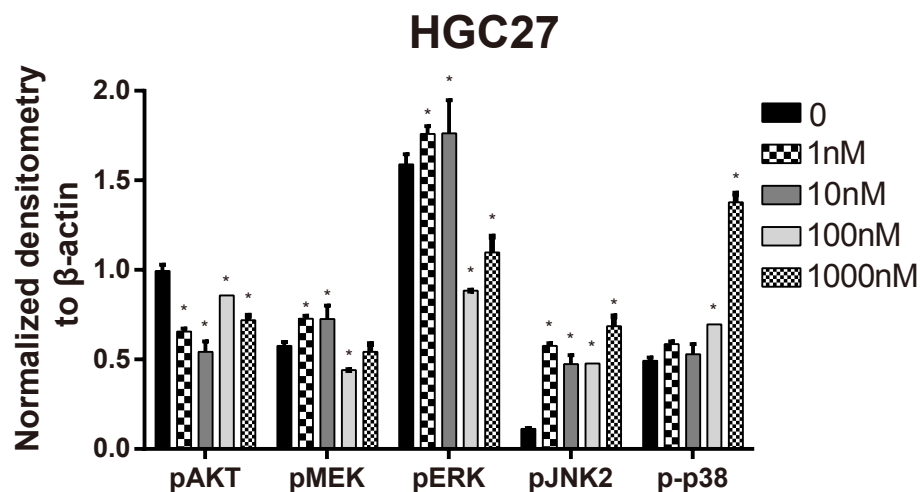

Supplement: Supplementary file 3 — Additional file 3: Figure S3. Gimatecan exerts antitumor activity via AKT and MAPK signaling pathways in HGC27 cells. (A) Gimatecan significantly inhibited the expression of TopI, pAKT, pMEK, and pERK, and activated the expression of p-p38 MAPK and pJUNK2 in HGC27 cells. Cells were starved in serum-free medium overnight, exposed to gimatecan or irinotecan for 48 h and harvested at 70–80% confluence. Total protein of HGC27 was extracted and the expression of TopI, pAKT, pMEK, pERK, p-p38 MAPK and pJNK2 were assessed by western-blotting followed by quantification and normalization by ImageJ. All data are means ± SD of three independent experiments. Compared with controls, *, p < 0.05. [file 12967_2017_1360_MOESM3_ESM.pdf]

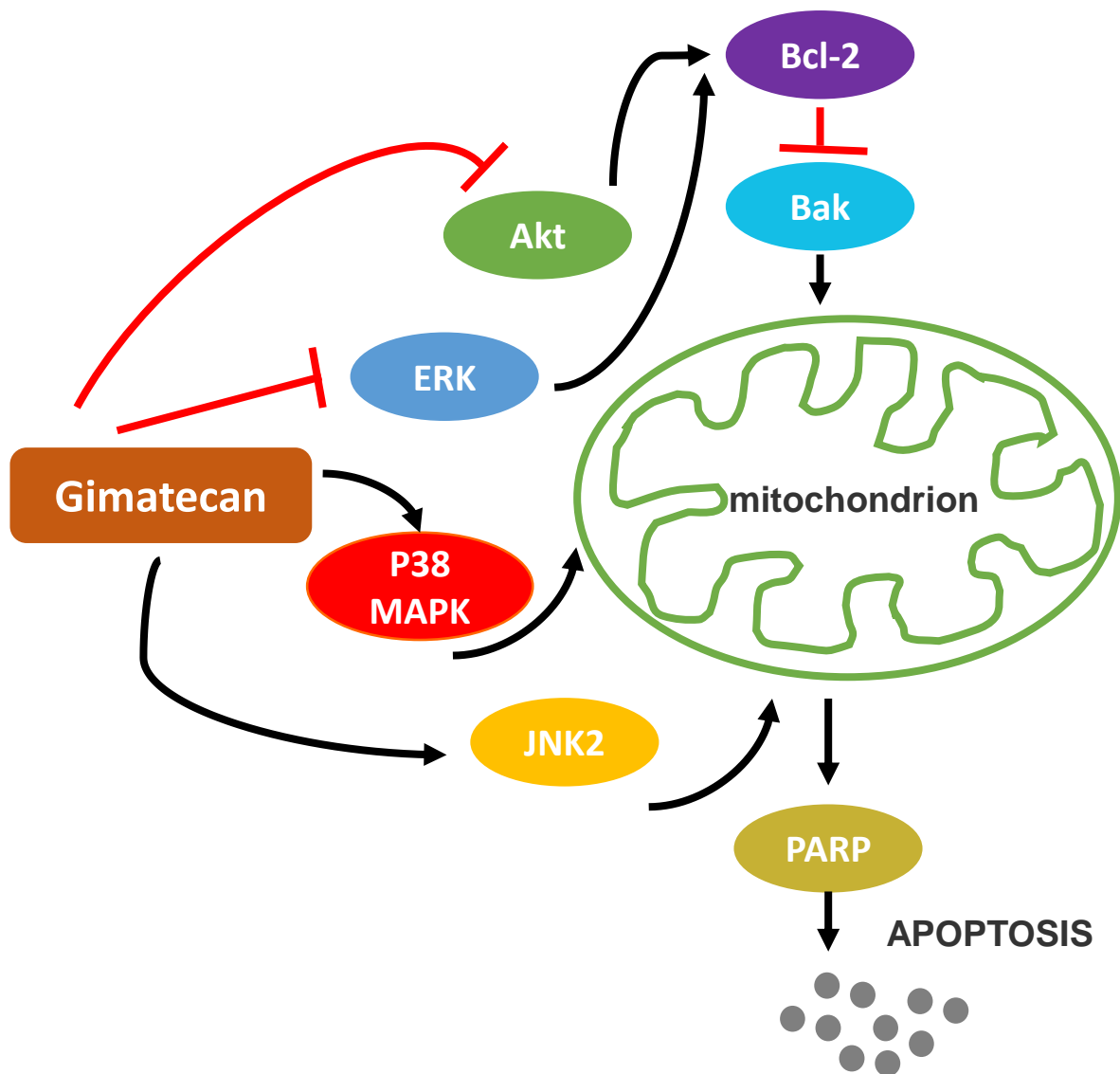

Supplement: Supplementary file 4 — Additional file 4: Figure S4. Schematic representation of proposed pro-apoptotic signaling pathways triggered by gimatecan in GC. [file 12967_2017_1360_MOESM4_ESM.pdf]
